# Measuring shared knowledge with group false memory

**DOI:** 10.1038/s41598-018-28347-4

**Published:** 2018-07-04

**Authors:** Yoshiko Arima, Ryoji Yukihiro, Yosuke Hattori

**Affiliations:** Department of Psychology, Kyoto Gakuen University, 1-1 Nanjo-ootani, Sogabecho, Kameoka, Kyoto 621-8555 Japan

## Abstract

We conducted two experiments to investigate the effects of word list consistency and group collaboration on false memory. Using the DRM (Deese-Roediger-McDermott) paradigm, the first experiment (n = 121) examined false memory in a group; participants collaborated to select keywords using a between-subject condition of a consistent or randomized word list. The proportion of false responses was larger than that of error responses, especially for the consistent word list condition (p < 0.001), and group collaboration increased false recognition for both word lists (p < 0.001). Using an applied within-subject word list condition, the second experiment (n = 119) confirmed the results of Experiment 1. Furthermore, individual differences Euclidean distance model analysis revealed a difference in the shared cognitive dimension related to group false memory.

## Introduction

This study explored the relationship between shared knowledge structure and group false memory. False memory is the recall or recognition of phenomena that did not occur. Based on a study by Deese^1^, which showed that people sometimes recalled words that were not present in but were associated with word lists, Roediger and McDermott^2^ determined an experimental paradigm of false memory called the DRM (Deese-Roediger-McDermott). A basic assumption of the present study is that false memory in a group can be an index of shared cognition because it occurs only if all group members fail to detect an error.

Group information processing studies have suggested that individual judgment is biased by schema stemming from an individual’s knowledge structure, and if the bias is shared in a group, the group process will exaggerate it^3–5^. However, determining what information is shared is difficult because each group forms a different shared cognition for each separate occasion. Regarding this problem, we can expect that group false memory provides a measurement of shared cognition. The purposes of this study were to investigate the conditions under which collaborative groups produced false memories and to explore the relationship between group false memory and shared knowledge structure.

## False memory

The basic DRM procedure is as follows. Participants take a free recall test after learning a word list associated with a certain non-presented word (critical word; CW) that is not included in the word list itself. After several recall tests, the participants take a recognition test that includes the presented associative words (targets), non-presented associative words (critical words; a response to critical words represents a false memory), and non-presented irrelevant words (distractors; a response to distractors represents an error). In the DRM paradigm, the proportion of false memory is usually much higher than that of errors.

Roediger III *et al*.^6^ suggested that automatic spreading activation in an associative-semantic network produces false memories. The activation-monitoring hypothesis^6^ predicts that false memory will occur when critical words receive cumulative indirect activation to a point where they can be interpreted as a memory. For the evidence of this hypothesis, false recognition increased and accurate recognition decreased with the number of targets in a word list. However, there are also evidences show contradictory. For instance, the forward association strength (recall rate of critical words with retrieval cues of the targets) does not correlate with false recognition, although the backward association strength (recall rate of targets with the retrieval cues of the CW) correlates with false recall. Brainerd and Wright^7^ argued that the forward and backward association strengths had independent effects, with the latter effects larger and more consistent. The main false memory process begins at the encoding stage^6,8^; adding to this effect, the retrieval process seems to have a small influence on false memory.

Based on the activation-monitoring hypothesis, we assume that false memory depends on the strength of the critical word as a semantic cue at the moment of encoding. In this article, ‘knowledge structure’ refers to the associative-semantic network related to certain cues. ‘Shared knowledge structure’ refers to the intersection of group members’ knowledge structures.

Studies have produced inconsistent results regarding group false memory. Several studies have shown that groups commit less than or at most the same number of false recalls as an individual^9–11^. In contrast, some evidence has shown that collaborative groups falsely recognize more critical words from categorized word lists than individuals^12,13^. These inconsistent results for group false memory can be explained by the properties of group memory.

## Group memory

Collaborative groups are known to exhibit a higher level of memory accuracy than individuals^5^. According to Hinsz^14^, groups are a better source of accuracy due to three factors: a larger information pool, the opportunity to revise errors, and efficient decision-making. Clark *et al*.^15^ tested whether the superiority of group memory was simply due to the efficiency of the larger information pool or to cooperative behavior. In their research, the results of groups of three people cooperating were more accurate than the results predicted by majority rule. Hence, from the perspective of group information studies, groups are expected to produce fewer false memories than individuals.

Despite their superior accuracy, collaborative groups tend to commit implicational errors in recall tasks. Some studies have reported that people are more confident about the accuracy of memories recalled by a group than that of memories recalled by individuals, even in cases where these memories are false. By giving approval and positive feedback to members, groups tend to exhibit greater response bias in their memories^16,17^. This is implicational error. Peker and Tekcan^18^ reported that groups committed false recall more often among friends than among non-friends. The authors argued that friend groups shared cognitive styles which caused social contagion of false memory^19^.

Combining the evidence of group accuracy and implicational errors, we can postulate that group false memory occurs when all group members fail to detect errors. It is worth noting that word lists should be prepared before matching with each society’s language and culture. Roediger and McDermott^2^ provided word lists from previously conducted free recall tests, but the non-native speakers did not produce the same proportion of false response as native speakers^20^. The robustness of the DRM experimental paradigm depends on the stability of the pre-stored shared knowledge structure in each culture. Operationally, the proportion of group false memory will depend on the consistency of the word lists with the participants’ pre-stored knowledge structures. The main purpose of the present article is to examine this hypothesis.

Little evidence has demonstrated that group remembering requires a shared knowledge structure. However, research on collaboration inhibition suggests this possibility. Collaborative inhibition is a phenomenon that group remembering cannot achieve nominal group; the hypothetical maximum summation of individual memories^21,22^. Weldon and Bellinger^22^ found that groups could recall better than individuals but not as well as nominal groups. Collaborative inhibition has been attributed to retrieval disruption caused by differences in retrieval organization among group members. Participants tend to categorize words in their unique individual style (subjective organization), and the differences among subjective organizations are related to collaborative inhibition. For example, Finlay *et al*.^23^ showed that participants learned items in the same order so that their retrieval strategies were more similar, resulting in less collaborative inhibition. Moreover, when the numbers of categories on a word list increased, group remembering was disturbed^12,24^. These discussions suggest that collaborative inhibition decreases when groups share their knowledge structure, as has been hypothesized for transactive memory^25^.

In contrast, Congleton and Rajaram^26^ found that collaborative inhibition increased with the sharedness of the knowledge structure. In their experiments, group members were asked to recall words individually after engaging in collaborative remembering of word lists from which categorical cues were excluded. The index of sharedness in a group was the similarity of the word order in individual free recall. The results showed that greater similarity of the order of the recalled words resulted in a smaller number of recalled than that in nominal groups. This result suggested that when the word lists lacked categorical cues, groups managed to share their knowledge structure in exchange for increasing memories.

Soro *et al*.^27^ examined false memory caused by ad-hoc categories (inconsistent with pre-stored knowledge but categorized by new associations) and found that ad-hoc categories produced false memories at a proportion of 0.33 to 0.37. These proportions were not as high as those of the false memories produced by pre-established categories (0.61), but this study proved that participants could find categorical cues from new associations. We explore whether a group also produces false memories with new associations from inconsistent word lists.

## Overview

Considering the findings from previous studies, we hypothesize that group collaboration increases false memories if a word list is arranged to be consistent with pre-stored shared knowledge. The main purpose of the first experiment was to examine the effect of the consistency of a word list with a pre-stored (before collaboration) shared knowledge structure on group false memory.

The degree of the consistency of the word list with the pre-stored knowledge structure was manipulated by consistent and randomized word list conditions. Fujita *et al*.^28^ have developed two types of word lists for Japanese individuals: blocked (consistent) word lists, which are equivalent to the word list in the DRM paradigm, and randomized word lists, which are randomized words across these consistent word lists (see Appendix A, which shows the word lists for Experiment 1). For the consistent condition, six word lists each had a critical word; for the randomized condition, each word list had no critical word. All words were the same between the two conditions. Yukihiro *et al*.^29^ found that participants produced more false recognition with consistent word lists than with randomized word lists. This result suggests that the effect of spreading activation provoked during the encoding process continues into the retrieval process on the recognition test.

We added the group collaboration condition to this word list condition. Collaborative groups were instructed to find six keywords (critical words), each of which should be associated with each of the six word lists. Participants completed the same recognition test individually before (pre-test) and after (post-test) group collaboration. The difference between the pre- and post-tests is the index of the effect of the group condition.

We hypothesized that the word list condition would affect both the pre- and post-test results. A consistent word list will activate an associative-semantic network that reaches critical words more often than a randomized word list, and the difference in the activation level will continue during the retrieval process. As a result, false recognition of consistent word lists will be larger than that of randomized word lists at the individual level. If a group shares an individual’s false memory, then the group will not be able to detect false recognition among its members. This situation will result in a higher ratio of false recognition of consistent word lists than of randomized word lists on post-test, whereas the error ratio will not increase after collaboration.

Hypothesis 1. Group false memory will be larger for the consistent word list condition than for the randomized word list condition.

Hypothesis 2. After collaboration, group false memory will increase more than errors, and the difference between the consistent and randomized word lists at the pre-test stage will remain the same.

In the first experiment, we also explored the effect of the number of critical words found as keywords by a collaborative group. If the social contagion process works in this experiment, the false recognition increment will be correlated with the number of critical words found by the collaborative groups.

The purpose of the second experiment was to confirm Hypotheses 1 and 2 using the within-subject condition for the word lists (Appendix B). In addition, the second experiment explored the post-shared (after group collaboration) knowledge structure measured with Multi-Dimension Scaling (MDS). Using MDS, we can generate a cognitive map of words that allows us to understand the knowledge structure graphically and analyze each groups’ weights on the dimensions of the cognitive map. Without specific hypotheses, the second experiment investigated the relationship between group false memory and the weights on the dimensions shared within a group.

## Experiment 1

### Method

#### Participants and design

The ethical committee of Kyoto Gakuen University approved this research (Research Number: 28-9), and all experiments were performed in accordance with the human research guidelines. Before performing the experiments, we informed participants about APA ethical standards for psychology and explained our research as follows:

Informed consent statements; “The purpose of this study is to compare individual and group memory. The benefits of the research will be a better understanding of group memory. The method that will be used to meet the purpose includes a test of your memory and a discussion group of four participants. If you feel uncomfortable with the procedure, you may withdraw from the study at any time. In that case, all information you provide will be erased from the data sets. You will not have face any repercussions in your course by withdrawing. After the experiments, we will provide an anonymous data set without revealing your ID number, gender, age or nationality. The data set will be used in writing a qualitative research article. Your identifying information will be kept anonymous through this research. If you agree to participate in this experiment, please fill in the blanks on the first page of the questionnaire”.

The first page of the questionnaire contained an informed consent sheet, which added the following sentences to the previous explanation: “Please fill in the blanks on the first page of the questionnaire. If you do not want to participate in this experiment, or you would like to participate without giving us your data, please do not fill in the blanks. By filling them in, you agree that you have read the instructions and are interested in participating in this study. If you have any questions or concerns after the experiments, please contact us at the following addresses”.

After the experiment, participants were provided an explanation and information on this study. The sample comprised 121 university students (73 males and 48 females, mean age = 19.47) in an experimental psychology class at Kyoto Gakuen University in Japan.

We used a 2 × 2 × 2 mixed design with a random effect (groups). The between-subject conditions were wordlist (consistent or randomized), while the within-subject conditions were time (pre or post-test) and response (false or error). Participants were randomly assigned to wordlist conditions.

#### Materials and procedure

Participants were instructed that they were going to participate in a psychological experiment about memory in an experimental psychology class. We conducted the same experiment four times in classes with approximately 30 students. Half of them were randomly assigned to the consistent or inconsistent wordlist condition and participated in the experiment. The other half were assigned to the other condition and waited in another classroom. The sequence of the assignment conditions (waiting first or experiment first) was counterbalanced.

Participants were seated apart from one another and handed a six-page booklet in which to write down the recalled words. The last page contained a recognition test. Participants were presented one of two wordlists – consistent or randomized – each containing six wordlists. The critical, non-presented words (CW) for the consistent wordlists were ‘rest,’ ‘read,’ ‘leg,’ ‘television,’ ‘happy,’ and ‘warm.’ The ‘consistent’ wordlist contained six lists consistent with a certain CW (the upper wordlist in Appendix A). The randomized wordlist contained six randomized words (the lower wordlist of Appendix A).

The 10 words on each wordlist were shown to participants sequentially for two seconds each on a screen that was set up in the front of the experimental room. After the 10 words were presented, the participants were given 90 seconds to recall and write them down. This procedure was repeated six times for the six wordlists. Subsequently, the participants were asked to complete the recognition test (pre-test), which contained 48 words, including the six CWs (false), 18 targets (correct), and 24 distractors (error). If participants checked the CW as the word in the wordlist on the recognition test, it was counted as a false response. If they checked the targets, it was counted as a correct response. If they checked the distractors, it was counted as an error response. The criterion of false memory was a significant difference between the proportion of false and error responses on recognition tests.

After the first recognition test, participants were divided into three- or four-person groups and seated apart from the other groups. There were 28 four-person groups and three three-person groups in total. The groups were instructed that there were six keywords for the six wordlists, and they were then asked to choose them from the recognition test page. Participants were told they should determine by themselves whether the keywords were contained in the wordlists they had seen before. After all groups finished their discussions, the participants were asked to take the second recognition test (post-test), which was the same as the first recognition test. In the second recognition test, participants were asked to answer individually – irrespective of whether the answer differed from their group’s answer. Each experiment took approximately 70 minutes. The participants were debriefed after the experiment at the end of the day, and two weeks later, they were given the results of the experiment with an explanation.

The dependent variables were the proportions of correct, false, and error responses. The proportion data were transferred using inverse sine transformation.

### Results and Discussion

#### Manipulation check: effect of wordlist on memory accuracy

To confirm the equivalency of the memorability of consistent and randomized wordlists, we examined the number of recalled and correct responses. We omitted incorrect recalls when counting recalled words. The mean total number of recalled words was 41.45 (SD = 5.73) in the consistent wordlist condition and 41.13 (SD = 5.88) in the randomized wordlist condition. There was no significant difference between the consistent and randomized wordlists (*F*(1, 119) = 0.09, *p* = 0.76, partial η^2^ = 0.001).

To test the effects of the conditions on correct responses, we conducted a 2 (wordlist: consistent or randomized) × 2 (time: pre or post-test) mixed-design ANOVA using groups as a random effect. It revealed that only the time condition affected correct responses (*F*(1, 114.99) = 7.46, *p* < 0.007, *r* = 0.25). While correct responses increased slightly after group collaboration (see Table 1), there was no significant difference in correct responses between the consistent and randomized wordlist conditions (*F*(1, 28.81) = 0.002, *p* = 0.97, *r* = 0.01).

**Table 1:** Means and SDs of recognition in each condition.

Condition	False	Correct	Incorrect
Consistent list			
Pre-test	53.21 (17.64)	71.91 (8.81)	8.97 (4.94)
Post-test	66.71 (13.28)	73.41 (9.57)	12.79 (8.83)
Inconsistent list			
Pre-test	45.14 (16.89)	70.95 (8.25)	11.75 (8.80)
Post-test	54.69 (17.01)	74.02 (9.27)	14.96 (10.36)
Total	55.07 (14.98)	72.73 (7.39)	12.06 (6.95)

#### Effect of group collaboration on false memory

Table 1 shows the means of each condition. We conducted a 2 (wordlist: consistent or randomized) × 2 (response: false or error) × 2 (time: pre or post-test) mixed-design ANOVA with a random effect (groups). It revealed a main effect of response, a main effect of time, and an interaction effect between response and time: *F*(1, 336.97) = 1793.57, *p* < 0.001, *r* = 0.92, *F*(1, 347.86) = 53.31, *p* < 0.001, r = 0.37, and *F*(1, 336.56) = 15.69, *p* < 0.001, *r* = 0.21, respectively. Using G*Power 3.1, we checked post-hoc power analysis for repeated measures within-between interaction. It yielded 2.63 as the critical F-values (n = 121, αerror power = 0.05, between = 2, within = 4, ICC: Internal correlation coefficients in pre-post false memory = 0.537).

There were more false responses than error responses on the pre-test, and false responses increased after group collaboration more than error responses did. Although the main effect of wordlist (the hypothesis 1) did not achieve significance (*F*[1, 28.87] = 3.89, p = 0.058, *r* = 0.35), interaction effects were found between response and wordlist (*F*[1, 336.56] = 38.38, *p* < 0.001, *r* = 0.32). There was no effect of gender difference (male or female) when it was added as a factor to the mixed-design ANOVA.

**Figure 1:**
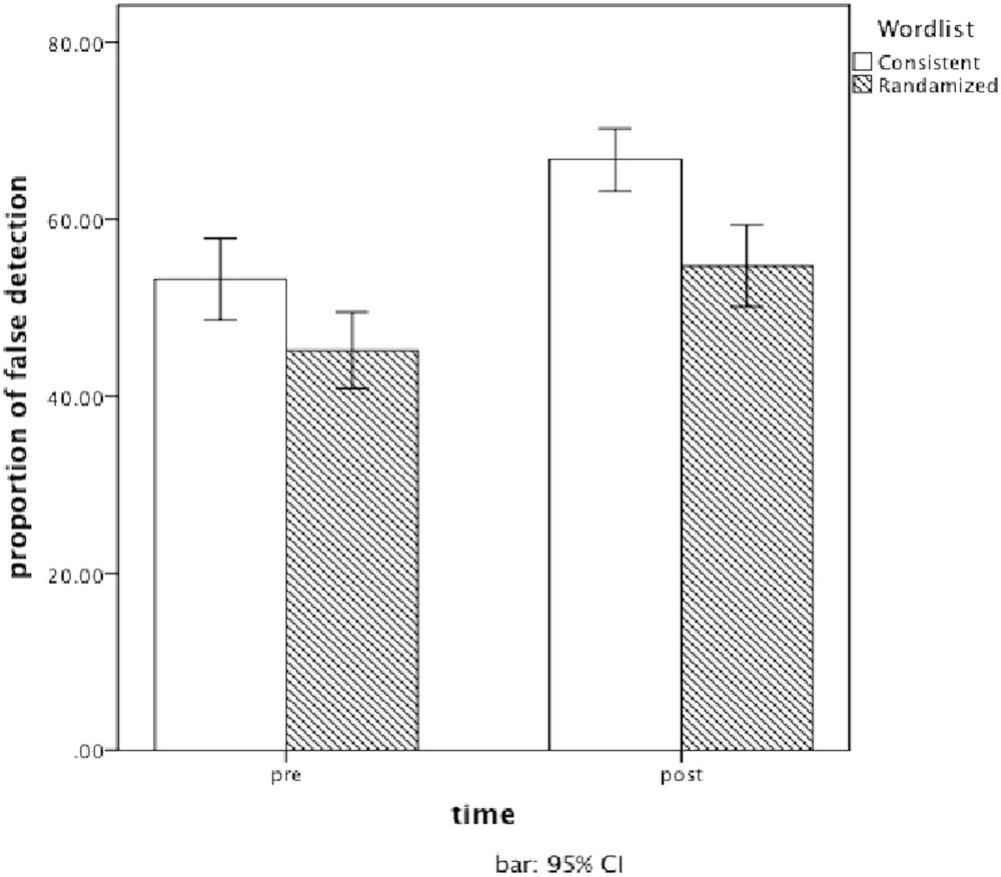
The effect of group collaboration for consistent and randomized wordlists in Experiment 1. White bars represent the mean proportion of false responses from pre-group collaboration to post-group collaboration in the consistent wordlist condition. Gray bars represent that in the randomized wordlist condition. Each bar represents the 95% confidence interval for each condition. In the two conditions, false memory increased from pre- to post-group collaboration at almost the same rate.

The multiple comparison tests showed that between the consistent and randomized wordlists, the difference in false responses was significant (*F*[1, 45.59] = 20.92, *p* < 0.001, *r* = 0.56), but the difference in error responses was not significant (F[1, 45.59] = 1.13, *p* = 0.294, *r* = 0.16). There was no interaction effect between wordlist and time (*F*[1, 347.86] = 1.46, *p* = 0.228, *r* = 0.16). As indicated in Fig. 1, false memory increased, maintaining the difference between the consistent and randomized wordlists before and after group collaboration. Thus, the hypothesis 2 was supported.

#### Effects of keywords on false and error responses

To test the social contagion hypothesis, we examined the effect of keywords found in a group on false or error responses. If false/error responses increased with the number of CWs/distractors selected as keywords, we determined that social contagion during group collaboration had an effect on individual memory.

As for keywords, the collaborative groups selected 0 to five CWs (*M* = 1.60, *SD* = 1.38) and 0 to five distractors (*M* = 1.0, *SD* = 1.62) from the recognition test sheet. Using these numbers of CWs and distractors as covariates, we conducted a 2 (wordlist: consistent or randomized) × 2 (response: false or error) × 2 (time: pre or post-test) mixed-design ANOVA with a random effect (groups). It revealed a main effect of response, a main effect of time, and an interaction effect between response and time: *F*(1, 328.75) = 382.4, *p* < 0.001, *r* = 0.54, *F*(1, 331.32) = 10.32, *p* = 0.002, *r* = 0.03, and *F*(1, 328.5) = 4.81, *p* = 0.029, *r* = 0.01, respectively. Both numbers of CWs and distractors in the keywords did not have any effect on false or error responses.

## Experiment 2

The purpose of Experiment 2 was to confirm the results of Experiment 1. The word list condition in Experiment 1 was the between-subject condition. In Experiment 2, the consistent and randomized word lists were set as the within-subject condition to confirm that the effect of word list consistency was irrelevant to the meaning of the word lists. The word lists (sets A and B; see Appendix B) were provided as a counterbalanced condition. Set A consisted of Lists 1, 3, and 5 from the consistent word lists and Lists 2, 4, and 6 from the randomized word lists in Experiment 1. Set B consisted of Lists 2, 4, and 6 from the consistent word lists and Lists 1, 3, and 5 from the randomized word lists in Experiment 1.

The results of Experiment 1 showed that group collaboration increased false memory for both the consistent and randomized word lists, although social contagion did not cause this increase. To investigate the reason why group collaboration increased false responses, Experiment 2 explored the post-shared knowledge structure using MDS and calculated individual difference evaluations for each word.

MDS is a method used to create a cognitive map from a distance matrix among objects × objects. In the present study, the objects were the words on the recognition test, and the distance matrix was calculated by individual evaluation (pleasure – not pleasure) for each word on the post-recognition test. If the data matrix is a three-way matrix (objects × objects × participants), Individual Differences Multidimensional Scaling (INDSCAL) can produce individual weights for each dimension using a weighted Euclidean model. INDSCAL provides information that enables understanding of the cognitive map and weight on each dimension in groups who have more false memories compared with those who have fewer false memories.

ALSCAL, which is an alternating least-squares algorithm, is a nonmetric MDS method adapted for INDSCAL^30^. Nonmetric MDS calculates disparity (quasi-distance) to maintain the same rank order as the raw data as much as possible. Stress is the fitness index for nonmetric MDS, which represents a monotone increasing function between distances on a cognitive map and distances on the raw data. This index should be under 0.20^31^.

As an additional analysis, we examined the validity of the first dimension (pleasure – not pleasure) of the INDSCAL with the external criterion obtained from the other experiment, in which the participants could evaluate the pleasantness of the targets. The participants in Experiment 2 simultaneously participated in an affect misattribution procedure (AMP) experiment^32^. The main purpose of our AMP experiment was to compare the effects of Japanese and Chinese Kanji characters among Japanese participants. The Japanese and Chinese Kanji characters were counterbalanced, and thus we could utilize the data on Japanese Kanji characters that appeared on the recognition test in the present study. The procedure and results of the AMP experiment are described in the Additional Analysis section.

### Method

#### Participants and design

The participants included 119 university students (83 males and 36 females, mean age = 19.87) in an experimental psychology class at Kyoto Gakuen University in Japan. We used a 2 × 2 mixed-design ANOVA with a random effect (groups); the within-subject condition was wordlist (consistent or randomized), and the within-subject condition was time (pre or post-test). The wordlist was counterbalanced using Set A/B conditions (see Appendix), to which participants were randomly assigned.

#### Materials and procedure

The procedure of Experiment 2 was the same as that of Experiment 1, except for the following. Half of the participants were randomly assigned to this experiment, and the rest were assigned to the AMP experiment to allow us to check the validity of INDSCAL. There were 29 four-person groups and one three-person group in total. The post-test was the same as the pre-test, but we added instructions to evaluate all words on a 5-point scale from 1 = not at all pleasant to 5 = very pleasant. Each experiment took approximately 80 minutes. The dependent variables were false responses for the consistent and randomized wordlists.

### Results and Discussion

#### Manipulation check

The effect of the Set A/B condition on total recall as well as false, correct, and error responses on the pre-test were checked for the counterbalance condition. Although total recall and error responses did not differ significantly, false and correct responses for the consistent wordlist were larger in Set A than in Set B: *F*(1, 117) = 5.821, *p* = 0.017, partial η^2^ = 0.05, *F*(1, 117) = 9.359, *p* = 0.003, partial η^2^ = 0.07, respectively. There were no significant differences in the randomized wordlist between Sets A and B. Considering these differences, the Set A/B condition was introduced for the following analysis.

#### Effects of group collaboration on false memory

To test the effects of conditions on false responses, we conducted a 2 (wordlist: consistent or randomized) × 2 (time: pre or post-test) × 2 (set: A or B) mixed-design ANOVA using a random effect (groups). It revealed a main effect of wordlist (*F*[1, 351] = 21.09, p < 0.001, *r* = 0.24) and time (*F*[1, 351] = 22.83, *p* < 0.001, *r* = 0.25) and interaction effects between wordlist and set (*F*[1, 351] = 6.92, *p* = 0.009, *r* = 0.14). The experiment 2 supported the hypothesis 1. Using G*Power 3.1, we checked post-hoc power analysis for repeated measures within-between interaction. It yielded 2.63 as the critical F-values (n = 119, αerror power = 0.05, between = 2, within = 4, ICC: Internal correlation coefficients in pre-post false memory = 0.45).

When we added gender differences (male or female) as a factor to this ANOVA, there was an interaction effect between gender and set (*F* [1, 115] = 9.05, *p* = 0.003, *r* = 0.27). The number of false memories was lower for females for Set B. There were no other effects related to sex difference. The proportion of false memories for females and males was not significantly different for Sets A and B (for Set A: male = 42, female = 20, for Set B: male = 40, female = 12). We will reexamine this gender difference in the MDS analysis.

**Figure 2:**
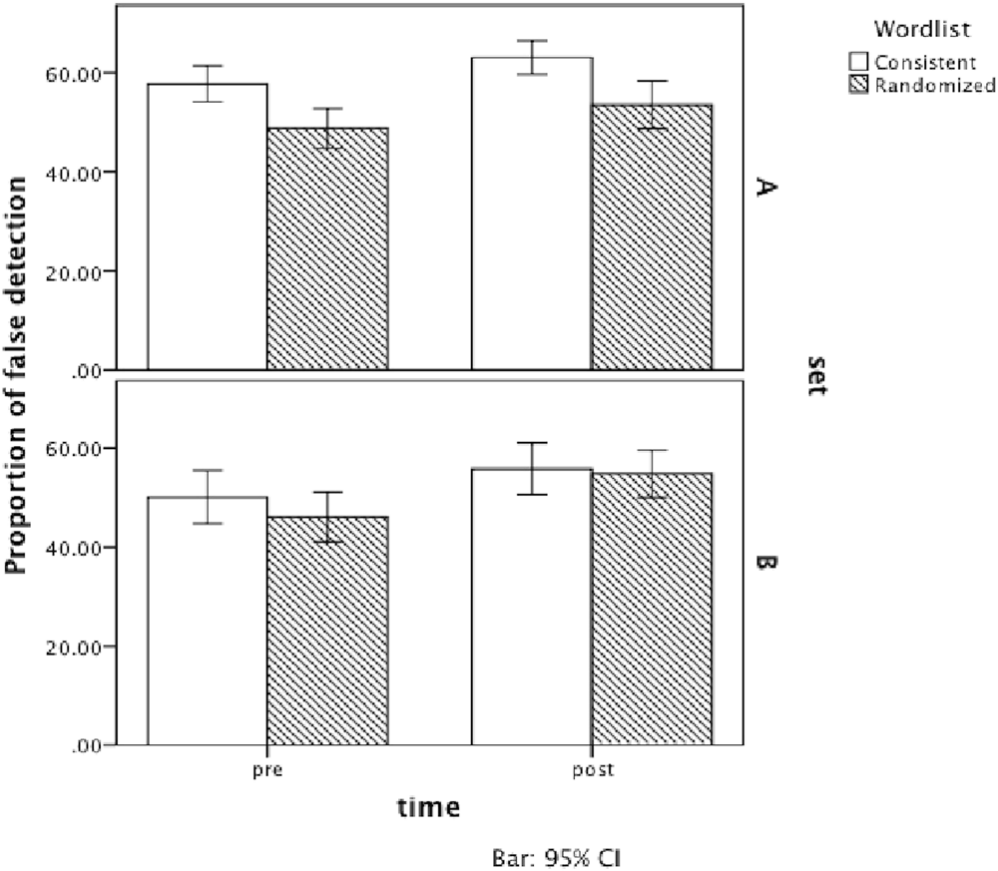
The effect of group collaboration for consistent and randomized wordlists in Experiment 2. White bars represent the mean proportion of false responses in the consistent wordlist condition. Gray bars represent that in the randomized wordlist condition. Because of the existence of an interactional effect between Sets A/B and the wordlist condition, the figures are depicted for Set (A) (upper) and Set (B) (lower). Multiple comparison tests revealed that there were no significant differences between the consistent and randomized wordlists for Set B, especially on the post-test. Nonetheless, it confirmed almost significant increments from pre- to post-group collaboration for all conditions.

Figure 2 depicts the results. A multiple comparison test revealed a difference in false memory between Sets A and B for consistent wordlists, *F*(1, 193.64) = 8.03, *p* = 0.005, *r* = 0.20, but not for randomized wordlists, *F*(1, 193.64) = 0.08, *p* = 0.78, *r* = 0.02. The consistent wordlist of Set A produced more false memories than that of Set B. The increments of false memory from pre- to post-group collaboration were significant for the consistent condition of Set A (*F*[1, 351] = 2.49, *p* = 0.03, *r* = 0.08), for the consistent condition of Set B (*F*[1, 351] = 2.64, *p* = 0.03, *r* = 0.09), and for the randomized condition of Set B (*F*[1, 351] = 2.64, *p* = 0.001, *r* = 0.09). For the randomized condition of Set A, there was slightly significant difference from pre- to post-group collaboration (*F*[1, 351] = 2.49, *p* = 0.058, *r* = 0.08). Generally, experiment 2 confirmed the results of Experiment 1. False responses for randomized wordlists and for consistent wordlists increased with group collaboration. The results of experiment 2 supported the hypothesis 1 and 2. However, the meaning of the words (Sets A/B) influenced the effects of the wordlists.

#### Keywords selected by collaborative groups

We checked the keywords selected during group collaboration that would affect the post-collaboration shared knowledge structure. The groups selected six keywords at one time, without arranging them to correspond with each wordlist. Table 2 shows the most selected keywords from the recognition test, which should contain a CW or target word. For Lists 1 to 4, most groups selected CWs as keywords. For List 5, most groups selected ‘memory’ as the keyword, and this was the correct response on that list. For List 6, most of the Set A groups selected ‘spring’ (correct response), and most of the Set B groups selected ‘warm’ (CW).

**Table 2:** Most selected prototype words and % of groups that selected the word.

List 1	List 2	List 3	List 4	List 5	List 6	warm
CW	rest	read	leg	television	happy	
**Set A**						
Consistent	rest		leg		memory	
	31.7%		55.6%		42.9%	
Randomized		read		television		spring
		30.2%		36.5%		57.1%
**Set B**						
Consistent		read		television		warm
		42.9%		50.0%		35.7%
Randomized	rest		leg		memory	
	21.4%		35.7%		35.7%	

#### ALSCAL for the evaluation score on each word

To apply ALSCAL for evaluations of the 24 words in the recognition test, four cases that had missing values were omitted, resulting in a balance of 114 participants. The 24 words were composed of a CW and three correct responses for each wordlist.

**Figure 3:**
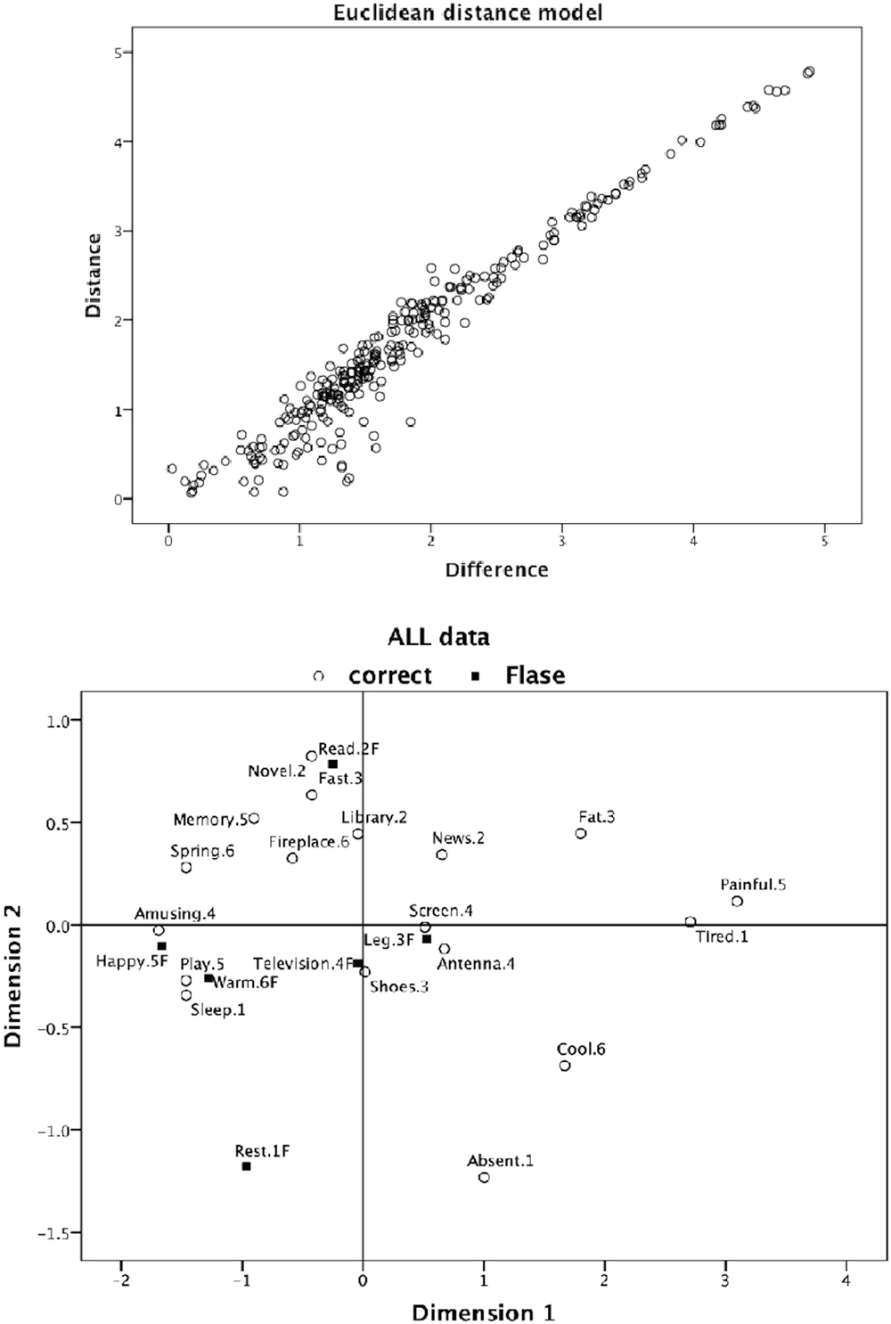
ALSCAL for all data and their fitness for the linear model. The vertical axis of the upper graph represents distances among words on the cognitive map (the lower graph), and the horizontal axis represents the order relationship of words in the raw data matrix. The dots on the map show a mono-linear increment relation between distance in the cognitive map and distance in the raw data.

**Figure 4:**
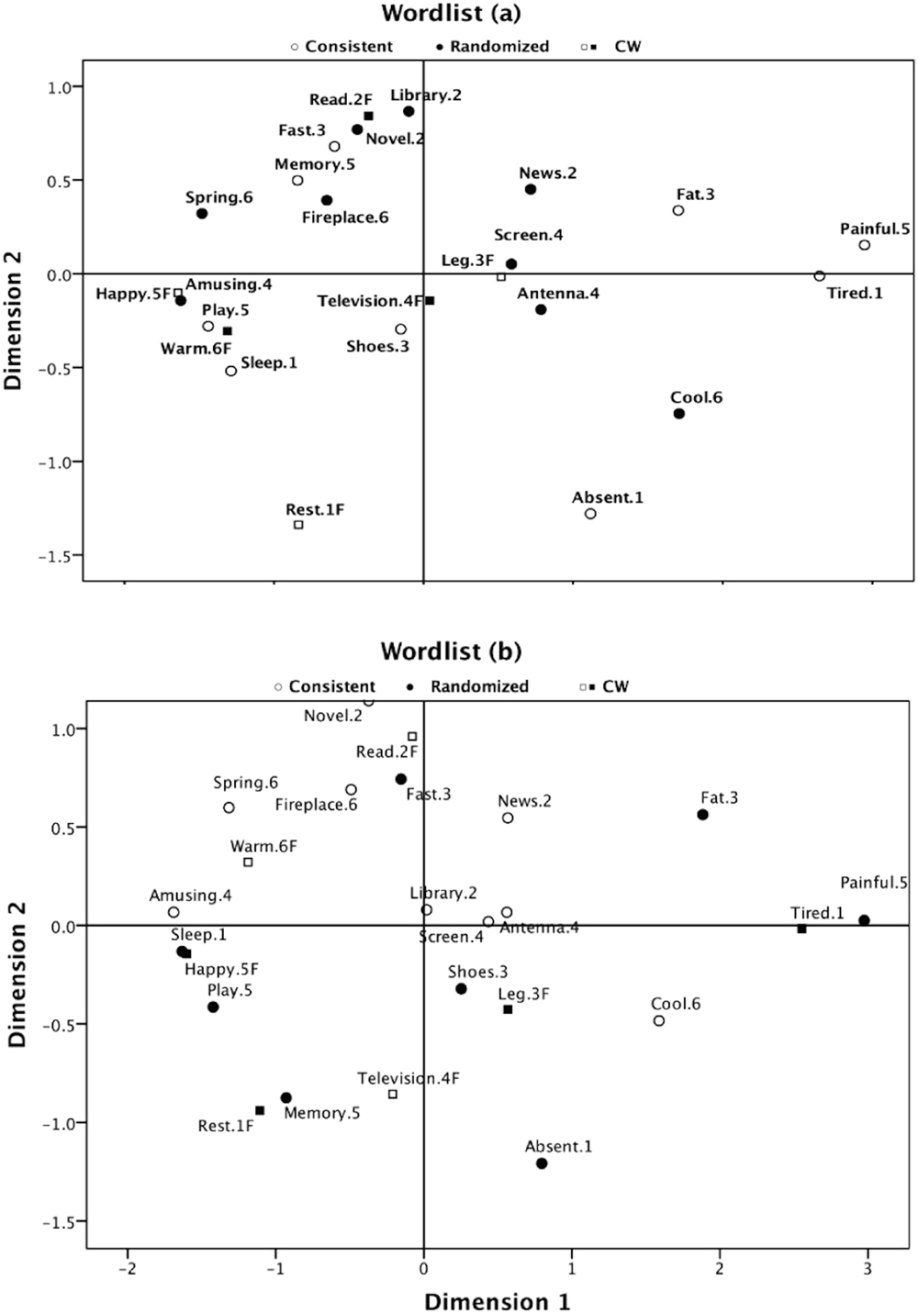
ALSCAL for each wordlist condition. The upper cognitive map is for the Set (a) wordlist, and the lower is for Set (b). The meaning of the horizontal dimension and the vertical dimension are almost the same as that for all data (Fig. 3); however, the positions of several words are different, especially on the second dimension.

Figure 3 shows the ALSCAL results for all data (S-stress = 0.13, squared correlation (RSQ) = 0.94). The horizontal dimension represents the *pleasure* or *not-pleasure* scales. The vertical dimension seems to discriminate between *intellectual activities* or *rest*. Considering the differences between Sets A and B, ALSCAL was conducted for the Set A and Set B conditions. ALSCAL yielded a two-dimension matrix for Set A (S-stress = 0.13, RSQ = 0.94) and for Set B (S-Stress = 0.17, RSQ = 0.91). Figure 4 shows the results. For the first dimension, the words were in almost the same position, regardless of whether they were from a consistent or randomized wordlist. For the second dimension, there were differences in the positions between Set A and Set B. For instance, while the word ‘memory’, which was selected as a keyword by most groups, was positioned in the plus direction on the second dimension in Set A, it appeared in the opposite position in Set B. ‘Memory’ seemed to be associated with leisure in Set B and with intellectual activity in Set A. This result suggested that participants shared a slightly different knowledge structure between Set A and Set B.

As we observed in the ANOVA results, the gender × set interaction effect on false memory was significant. This effect seemed to be caused by the difference in knowledge structure between males and females. Females’ cognitive map was more closely resembled with Set B than Set A, while males’ cognitive map was more closely resembled with Set A than Set B, especially for the position of ‘memory’ (see Appendix C).

#### Effects of shared knowledge structure on false memory

Based on the ALSCAL for all data (stress = 0.13, squared RSQ = 0.94; see Fig. 3), INDSCAL provided group weights for the first and second dimensions. We used group weights to investigate the influence of group cognition on false memory. Using group-level data (*n* = 30), we conducted an ANCOVA (wordlist: consistent or randomized) × 2 (time: pre or post-test) × 2 (set: [A] or [B]) with the covariate of group weight for the first and second dimensions. It yielded a two-way interaction between word and time (*F*(1, 26) = 4.96, *p* = 0.035, partial η^2^ = 0.16) and three-way interactions among wordlist × time × group weight for the second dimension (*F*(1, 26) = 12.74, *p* = 0.001, partial η^2^ = 0.33) and wordlist × time × set (*F*(1, 26) = 6.12, *p* = 0.02, partial η^2^ = 0.19). Using G*Power 3.1, we checked post-hoc power analysis for repeated measures within-between interaction. It yielded 2.71 as the critical F-values (n = 30, αerror power = 0.05, between = 2, within = 4, ICC: Internal correlation coefficients in pre-post false memory = 0.49).

**Figure 5:**
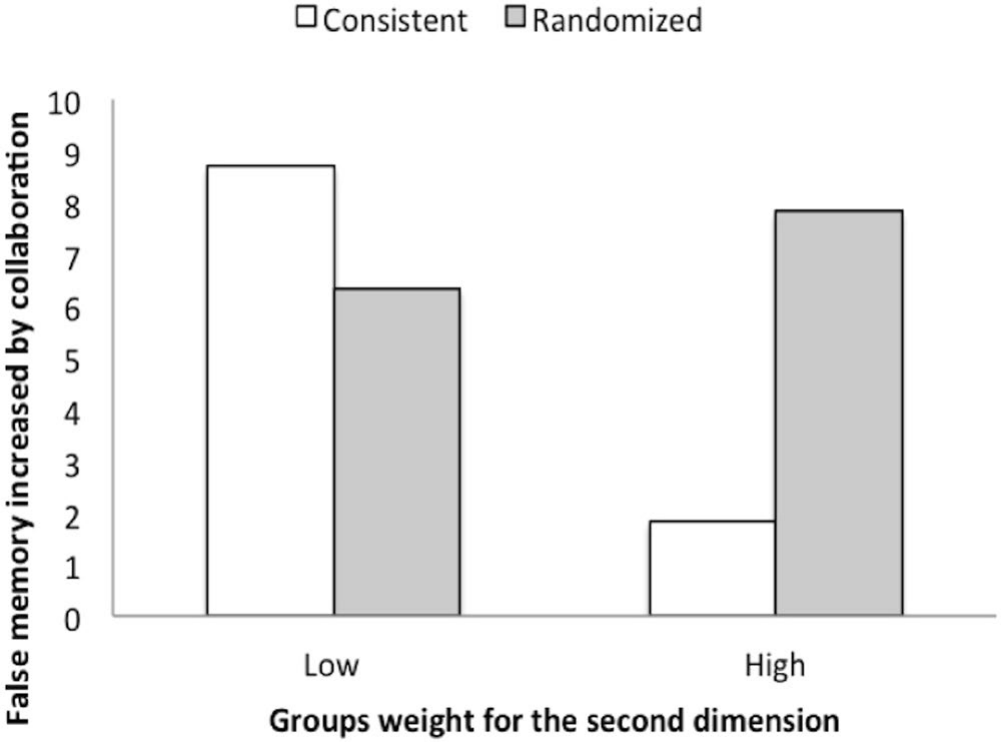
Effect of ALSCAL dimension on increment of false memory. The vertical index is the difference between pre- and post-collaboration false memories. The groups were divided into two levels weight for the second dimension produced by INDSCAL. The high groups were those with higher weights than the mean weight of the second dimension, and the low groups were those with lower weights. While the increment of the consistent wordlist decreased with the weight for the second dimension, that of the randomized wordlist increased with it.

As Fig. 5 shows, we found that the groups that had highly weighted the second dimension increased their false memory for the randomized wordlist instead of suppressing it for the consistent wordlist. This result suggested that the high weighting for the second dimension groups could rearrange randomized wordlists to find CWs.

### Additional analysis: test for validity of the INDSCAL

To test the validity of the dimensions yielded by the INDSCAL, we measured the participants’ preferences for Japanese Kanji characters using an AMP experiment conducted simultaneously. The Kanji characters used as targets in the AMP experiment were part of a word on the recognition test in Experiment 2. If the INDSCAL was valid, participants who were weighted on the first dimension (pleasure or not pleasure) would be more likely to discriminate Kanji characters on pleasantness than those who were not weighted on the first dimension of INDSCAL.

Participants were asked to respond with a *pleasure* or *no pleasure* key for each Kanji image on the monitor’s screen (see Appendix C). After 12 exercise trials, the participants responded to the Chinese Kanji targets block (36 trials) and the Japanese Kanji targets block (36 trials). In this article, we report the results of the Japanese Kanji block. The block order of the Japanese and Chinese conditions was counterbalanced by the random assignment of even or odd numbers to each participant. The sequence of the condition assignment (waiting first or experiment first) was also counterbalanced. The intrusion of memory from the Kanji characters to the recognition test was low because pronunciation was different between the single Kanji character and the word including the Kanji for Japanese. For example, the Japanese pronunciation of the word ‘amusing’ is ‘Tano-shii’, in which ‘Tano’ is represented by a Kanji character. The pronunciation of the Kanji’Tano’ changes to ‘Raku’ when Japanese individuals see the same Kanji as a single character.

**Figure 6:**
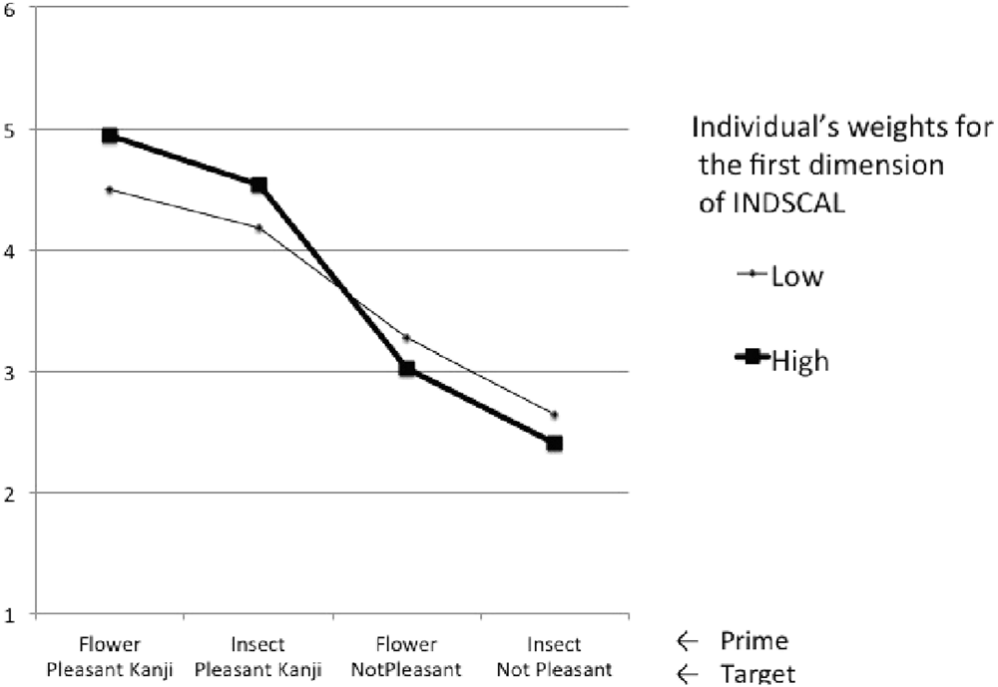
Response for ‘pleasure’ for Kanji characters used in the false memory experiment. The vertical index is number of responses for ‘pleasure’ for 12 Japanese Kanji, which appeared on the recognition test. Responses for pleasant Kanji caused pleasure responses more than responses for not-pleasant Kanji. The participants were divided into two levels of weight for the first dimension (pleasure or not-pleasure for the recognition test words) produced by INDSCAL. The difference between pleasant and not-pleasant Kanji for high-weight participants was greater than that for low-weight participants. The subliminal prime had the same effects on both participant groups.

Before presenting the Kanji targets, we presented subliminal priming stimuli as pleasant pictures (flowers) or unpleasant pictures (insects). The sequence of a trial was a 20-ms prime, a mask, a 500-ms target, and a mask. The last mask appeared until the participants responded. The presentation order of the Kanji targets was randomized by the Millisecond Program “Inquisit 3”. For the analysis, the 12 Japanese Kanji characters were divided into relatively pleasant (e.g., play or amusing) or relatively unpleasant (e.g., painful or tired) Kanji. As shown in Fig. 6, participants who were weighted on the first dimension of INDSCAL discriminated more clearly between pleasant and unpleasant Kanji than those who were not weighted on this dimension. The interaction effect between the Kanji condition and the individual weight for the first dimension was significant (*F*[1, 105] = 3.04, *p* = 0.032, partial η^2^ = 0.08). This result supported the validity of the first dimension of the INDSCAL.

## Discussion

Experiment 1 showed that participants tended to produce more false responses for the consistent word lists than for the randomized word lists. This effect continued after group collaboration. This result was confirmed by the experiment 2. Hence, this study supported hypothesis 1, which stated that group collaboration cannot detect false responses corresponding to the levels of activation evoked by the word lists. Moreover, as hypothesis 2 predicted, false responses increased after group collaboration, but error responses did not. Although the social contagion hypothesis was a plausible explanation for this result, the number of critical words in the keywords found in a group did not correlate with the number of false responses. Considering the fact that the correct responses also increased after group collaboration, we can interpret the increase of false memory as follows. Group members’ recognition words have an effect as a mutual stimulus for other members’ semantic associations^15^, resulting that group false memories increased only if they admitted those memories were correct. This process will explain the results of previous researches that group remembering could decrease collaborative inhibition if they shared subjective organization.

The purpose of Experiment 2 was to confirm the results of Experiment 1 using the within-subject condition of the word list. Experiment 2 confirmed that false memories increased after group collaboration irrespective of the meaning of the words, which maintained the difference between the consistent and randomized word lists. The results of experiment 1 and 2 supported the hypothesis 2.

However, an unexpected interaction effect with the word list was found in Experiment 2 for sets A/B, which were prepared as a counterbalance condition. Our exploration using ALSCAL suggested that the participants had a slightly different knowledge structure between sets A and B with the second dimension. The group weight for the second dimension had an effect on the increase in false memory for randomized word lists. From these observations, we could conclude that increase of group false memory is related to the ad-hoc similarity in cognitive proximity among words rather than to the straightforward sharing of recalled words as hypothesized in social contagion. In some sense, the groups that shared the second dimension were intelligent enough to produce false memories from randomized wordlists.

There are two possibilities for shared cognitive processes in a group. The first possibility is the shared knowledge effect, in which group processes exaggerate shared cognitive schema and attenuate unshared cognitive schema^33^ (assimilation). The second possibility is the audience-tuning effect^34^, in which group processes accommodate unshared information to adapt to shared schema (accommodation). The results of this study imply that assimilation usually works in a group process. However, if inconsistent information is provided, the accommodation process works to create an ad-hoc knowledge structure.

Figure 5 shows an increase in false memories for randomized word lists accompanied by a decrease in false memories for consistent word lists. This process appears to be related to the finding that collaborative inhibition increases with group collaboration based on ad-hoc shared knowledge. A trade-off relationship may exist between creating post-shared knowledge and maintaining pre-stored shared knowledge.

Experiment 2 also revealed a gender effect on false memory as a gender × set (A/B) interaction and gender differences in the cognitive maps. Collective intelligence research found that females were better at reading others’ emotions in a collaborative group^35,36^. Females may be more flexible than males when a group requires an accommodation process. However, because the preference for each word was collected at the post-test and not at the pre-test, we could not determine whether a gender difference in cognitive maps existed before or after group collaboration in this study.

Group false memory is shaped by a shared knowledge structure. This process can have both positive and negative consequences. With shared knowledge, we can easily exchange concepts, but we also may pass on mistakes that we take for granted, such as with Fukushima’s nuclear accident. If a group has diverse knowledge structures among group members, the group will be able to detect mistakes; at the same time, such a group will experience difficulties due to miscommunications. To overcome this problem, we need a complexity in our shared knowledge structure.

However, a trade-off may exist between complexity in shared knowledge and the amount of shared memory. Further research on this problem is needed. Generally, the results of this study show that group false memory can be an index for a pre-stored and a post-shared knowledge structure. False memories in consistent word lists can be utilized as a measure to understand what associations are shared in pre-stored knowledge, and randomized word lists can reveal associations with which a group creates a post-shared knowledge. We expect group false memory will provide a measure to investigate further research.

### Data availability statement

The data that support the findings of this study are available from the corresponding author, Y.A. on request.

## Electronic supplementary material

Supplementary Information
