# Supplementary material for: Measuring shared knowledge with group false memory
Source: Sci Rep. 2018 Jul 4;8:10117. doi: 10.1038/s41598-018-28347-4 (PMC6031639; doi:10.1038/s41598-018-28347-4)

**Measuring shared knowledge with group false memory**

Yoshiko Arima* Ryoji Yukihiro Yosuke Hattori Kyoto Gakuen University

Correspondence concerning this article should be addressed to Yoshiko Arima, Faculty of Human and Cultural Studies, Kyoto Gakuen University, 1-1 Nanjo-ootani, Sogabecho, Kameoka, Kyoto, 621-8555, Japan. E-mails may be sent to [arima@arimalab.com](mailto:arima@arimalab.com).

Appendix A

Wordlist for Experiment 2 (Consistent and randomized as between-subject condition)

Consistent List 1 List 2 List 3 List 4 List 5 List 6

Critical word rest read leg television happy warm

Wordlist absent news shoes amusing play spring

vacation letter walk see travel cold

Sunday talk run radio music heater

sleep library fast screen memory cool

school voice hands drama picnic stove

holiday write step eye time summer

tired novel fat antenna painful fireplace

break book long program glad room

sick reading socks channel hiking fire

lesson journal slim movie sad futon

Randomized List 1 List 2 List 3 List 4 List 5 List 6

Wordlist absent news shoes amusing play spring

travel cold vacation letter walk see

run radio music heater Sunday talk

sleep library fast screen memory cool

picnic stove school voice hands drama

step eye time summer holiday write

tired novel thick antenna painful fireplace

glad room break book long program

socks channel hiking fire sick reading

lesson journal slim movie sad futon

Appendix B

Wordlist for Experiment 2 (Consistent and Randomized was within-subject condition)

Set A List 1 List 2 List 3 List 4 List 5 List 6

Consistent or Random C R C R C R

Critical word rest leg happy

Wordlist absent news shoes amusing play spring

vacation cold walk letter travel see

Sunday radio run heater music talk

sleep library fast screen memory cool

school stove hands voice picnic drama

holiday eye step summer time write

tired novel fat antenna painful fireplace

break room long book glad program

sick channel socks fire hiking reading

lesson journal slim movie sad futon

Set B List 1 List 2 List 3 List 4 List 5 List 6

Consistent or Random R C R C R C

Critical word read television warm

Wordlist absent news shoes amusing play spring

travel letter vacation see walk cold

run talk music radio Sunday heater

sleep library fast screen memory cool

picnic voice school drama hands stove

step write time eye holiday summer

tired novel thick antenna painful fireplace

glad book break program long room

socks reading hiking channel sick fire

lesson journal slim movie sad futon

Appendix C

Gender difference in knowledge structure


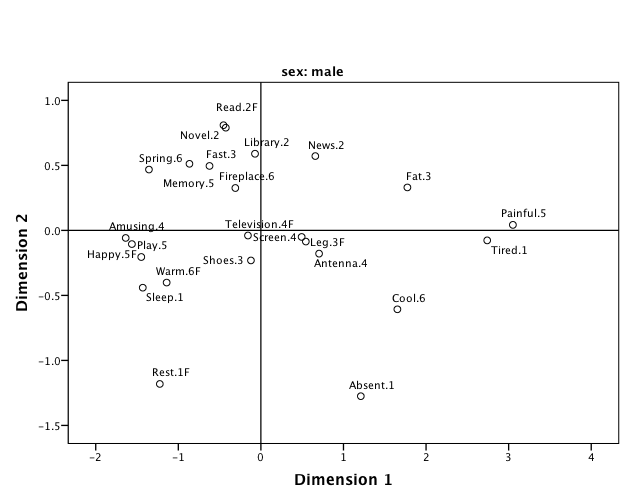


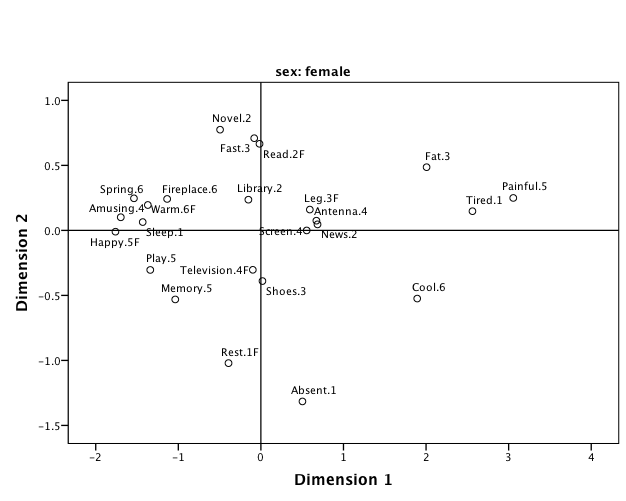


Male: S-Stress = .11, RSQ = .96

Female: S-Stress = .10, RSQ = .96

Caption: The first dimension was almost the same; however, the word position on the second dimension was slightly different, especially for ‘memory’.

Appendix D

Kanji characters used for AMP experiment

Japanese Characters（Pleasant to neutral）


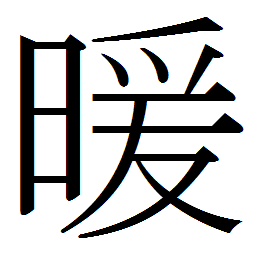

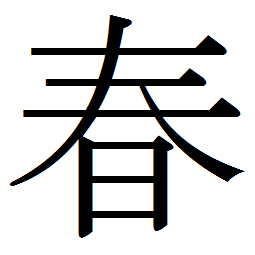

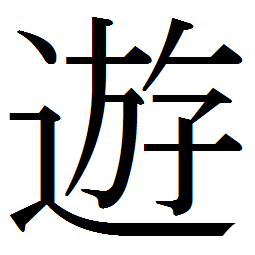

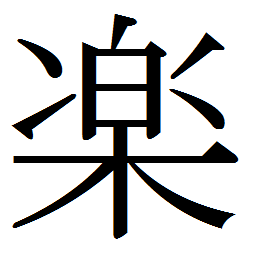

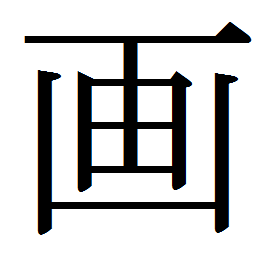

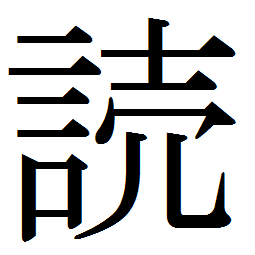


Warm, Spring, Play, Amusing, Screen, Read

Japanese Characters (Neutral to Unpleasant)


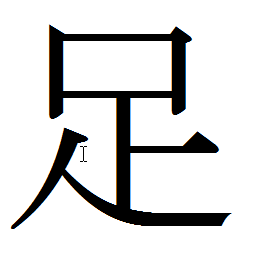

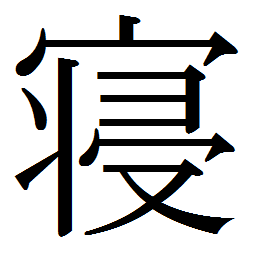

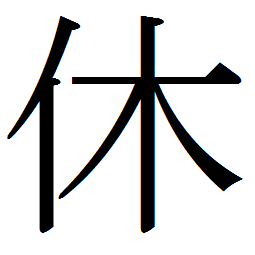

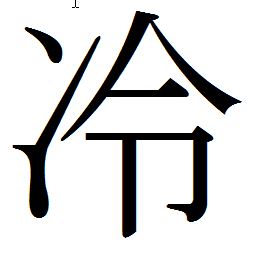

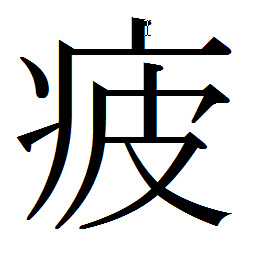

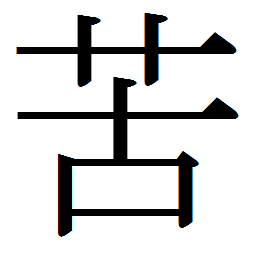


Leg, Sleep, Rest, Cold, Tired, Painful

Chinese Characters (Japanese cannot read them)


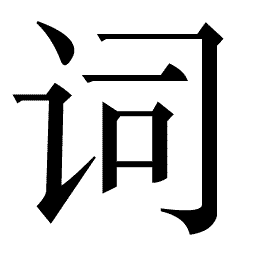

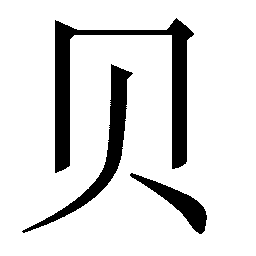

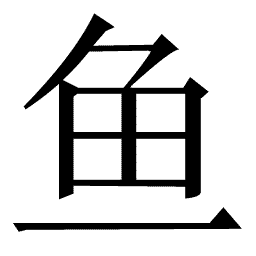

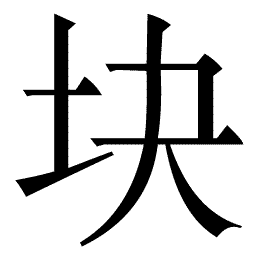

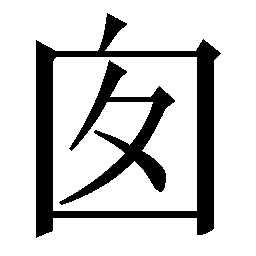

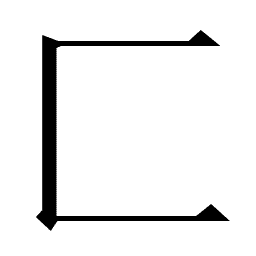


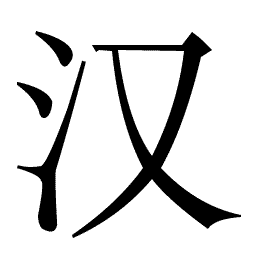

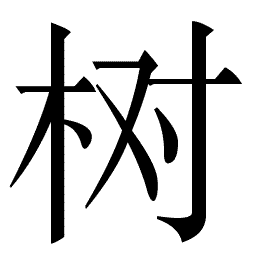

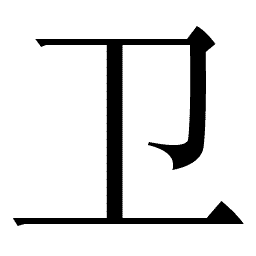

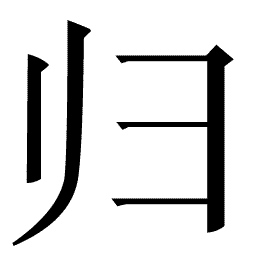

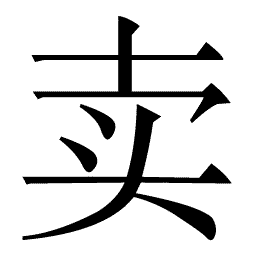

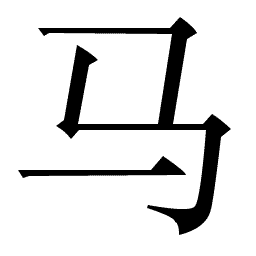

Supplement: Supplementary file 1 — Supplementary Information [file 41598_2018_28347_MOESM1_ESM.docx]
